# Presurgical Succinate MetAstatic Risk Tool (P-SMART) in Paragangliomas

**DOI:** 10.1007/s12022-025-09878-9

**Published:** 2025-09-30

**Authors:** Elena Rapizzi, Lorenzo Zanatta, Alice Santi, Fabio Staderini, Niccolò Galeotti, Tonino Ercolino, Francesca Amore, Clotilde Sparano, Mario Maggi, Letizia Canu

**Affiliations:** 1 Dept. Experimental and Clinical Medicine, University of Florence, Florence, Italy; 2 Centro Di Ricerca E Innovazione Sulle Patologie Surrenaliche, AOU Careggi, Florence, Italy; 3 ENS@T Centre of Excellence, Florence, Italy; 4 Dept. Experimental and Clinical Biomedical Sciences, University of Florence, Florence, Italy; 5 Careggi University Hospital, Florence, Italy

**Keywords:** Pheochromocytomas and paragangliomas, Prognostic markers, Succinate levels, Metastatic score, P-SMART

## Abstract

Paragangliomas (PGLs) are rare malignant non-epithelial neuroendocrine neoplasms characterized by a strong genetic determinism and heterogeneous metastatic potential with no reliable histopathological predictors. In this retrospective study we investigated the role of serum succinate as a biomarker for metastatic risk and developed a novel preoperative scoring tool. Seventy patients with PGLs evaluated between 2006 and 2023 were analysed. Clinical, biochemical, imaging, and genetic data were collected. Germline genetic variants were analysed via Sanger sequencing or NGS through a targeted panel of susceptibility genes. Serum succinate concentrations were quantified by gas chromatography–mass spectrometry. Succinate levels were significantly higher in patients with Cluster 1 genetic variants (p < 0.001), extra-adrenal PGLs (p = 0.006), and metastatic disease (p = 0.024). We developed a novel preoperative risk assessment tool, the P-SMART (Preoperative Succinate MetAstatic Risk Tool), combining serum succinate levels, tumour size, and location. P-SMART assigns: 3 points for extra-adrenal localization, 3.5 points for serum succinate ≥ 8.95 µM, and 3 points for tumour size ≥ 7.0 cm. In our cohort a P-SMART score > 4.75 predicted metastatic disease with 72.7% sensitivity and 83% specificity, outperforming the ASES score (Age, Size, Extra-adrenal, Secretory type; AUC 0.891 vs 0.752, p = 0.005). Though limited by sample size and retrospective design, our findings suggest that succinate is a minimally invasive biomarker that could enhance preoperative metastatic risk stratification, especially when integrated into a multiparametric score such as P-SMART. Larger prospective studies are needed to validate its role, but P-SMART could optimize clinical decision-making, refine patient selection for whole-body imaging, reduce unnecessary radiation exposure, and inform surveillance strategies.

**Supplementary Information:**

The online version contains supplementary material available at 10.1007/s12022-025-09878-9.

## Introduction

Paragangliomas (PGLs) are rare non-epithelial neuroendocrine neoplasms that arise from the adrenal medulla or extra-adrenal paraganglia. These lesions are characterized by strong genetic determinism and represent one of the best examples of heritable tumours [1]. Approximately 70% of PGLs are associated with germline or somatic mutations in more than 20 susceptibility genes identified to date [2, 3]. The remaining cases, according to the current evidence, seem to occur sporadically. These driver genes are categorized into three main molecular clusters: the pseudohypoxia cluster (Cluster 1), the kinase-signalling cluster (Cluster 2), and the Wnt-signalling cluster (Cluster 3). Cluster 1 includes pathogenic variants in Krebs cycle genes (*SDHB, SDHA, SDHC, SDHD, SDHAF2, FH, MDH2*, and *IDH1*) and in genes regulating hypoxia-inducible factors (HIF)−1α and/or HIF-2α (*VHL, EPAS1, EGLN1/2* and *DLST*). Cluster 2 is characterized by an activated kinase receptor profile and includes pathogenic variants in *RET, NF1, TMEM127, HRAS, MAX*, and *FGFR1*. Cluster 3 encompasses tumours with activated Wnt signalling, often associated with *MAML3* fusion genes or *CSDE1* pathogenic variants.

As stated in the 2017 WHO classification of endocrine tumors, and maintained in the 2022 WHO version, all PPGLs have been considered as malignant tumours with uncertain metastatic potential as there are no pathological features that reliably predict metastatic behaviour [4]. Many clinical, genetic, biochemical and pathological parameters have been reported to help estimate metastatic risk in PGLs. For example, thoracic and abdominal extra-adrenal PGLs are more frequently associated with metastasis [5]. Moreover, Cluster 1 pathogenetic variants, especially *SDHB*, have been associated with increased metastatic risk, whereas certain *RET* mutations appear to predict a lower one [6]. PGLs are normally, but not always, characterized by catecholamines synthesis and secretion [7]. Tumours producing noradrenaline and dopamine, given the early interruption of the catecholamine synthesis cascade, are considered less differentiated and carry a higher metastatic risk compared to adrenaline-producing tumours [8]. Finally, larger lesions [9], higher percentage of Ki67 staining [10], and more advanced stage, as per tumour-node-metastasis (TNM) staging [11], are more at risk for metastatic disease. However, none of the above-mentioned parameters alone is sufficient to correctly predict the presence of metastasis. Therefore, efforts have been made to develop scoring systems to accurately classify the malignancy risk. Most of the scores are dependent from histologic or genetic parameters, such as Pheochromocytoma of the Adrenal Gland Scaled Score (PASS) [12], Grading system for Adrenal Phaeochromocytoma and Paraganglioma (GAPP) [13], modified GAPP (m-GAPP) [14], COmposite Pheochromocytoma/paraganglioma Prognostic Score (COPPS) [15] and Size, Genetic, Age, and PASS (SGAP) [16] scores. Additionally, two scores have been built thus far to predict metastatic risk in the preoperative setting: age, size, extra-adrenal location, secretory type (ASES) [17] and gene variant, methoxytyramine, and size of tumour (GMS) [18]. The latter relies on genetic information, which is not always available at time of surgery. ASES score, instead, combines parameters to preoperatively predict the metastatic potential of PGLs. In this system, scores < 2 showed a negative predictive value of 96.5%. It is worth mentioning, that despite their use in individual practices is not discouraged, none of the histopathological risk scoring systems has been endorsed by the 2022 WHO classification.

In recent years, succinate has emerged as a promising biomarker for PGLs. Succinate is an intermediate metabolite of the Krebs cycle, generated from succinyl-CoA by succinyl-CoA synthetase and converted to fumarate by succinate dehydrogenase (SDH). PGLs harbouring *SDHx* mutations exhibit dysfunction of the SDH enzyme complex, leading to intracellular accumulation of succinate [19]. Accumulated succinate acts as an oncometabolite by inhibiting HIF-1α prolyl hydroxylases (PHD) and α-ketoglutarate-dependent dioxygenases, including histone and DNA demethylases [20, 21]. Notably, increased cytosolic succinate concentrations have been observed in multiple cancer types, not only in PGLs. Lamy et al. [22], moreover, demonstrated a correlation between succinate levels and disease extension in patients with *SDHB*-mutated PGL.

In this study, therefore, we aimed to investigate the reliability of succinate as a biomarker in PGLs and its potential utility, combined with other known risk factors, in identifying patients at higher risk of metastasis.

## Materials and Methods

### Study Design

Retrospective cross-sectional study.

### Study Population

We enrolled 70 outpatients affected by PGL evaluated at Endocrinology Unit of University Hospital of Florence between January 2006 and June 2023, and at least 18 months of follow up. All participants gave written informed consent for the enrolment (ENS@T protocol 59/11, version 1.3). The diagnosis of PGL was made by histology after surgical removal and/or imaging (CT or MRI and nuclear medicine exams such as [^68^ Ga] Ga-DOTA PET/CT, [^18^F] F-DOPA and [^131^I]MIBG). We collected all relevant information, including gender, age at diagnosis, size of the primary tumour, AJCC TNM stage at diagnosis, presence of metastases at last follow up, length of follow up, outputs of 24-h urinary metanephrine and normetanephrine.

### Genetic Analysis

A genetic test was performed in all patients to find any germline variants in DNA extracted from peripheral blood leucocytes. Before 2018, germline variants were analysed using Sanger sequencing of 9 susceptibility genes: *SDHA, SDHB, SDHC, SDHD*, *MAX, SDHAF2, TMEM127, VHL* and *RET.* From 2018 onward, variant analysis was conducted using Next Generation Sequencing (NGS) with a custom-targeted panel of 15 genes previously identified as drivers: *SDHA, SDHB, SDHC, SDHD, SDHAF2, VHL, MAX, TMEM127, RET, EPAS1, FH, EGLN1, KIF1Bβ, SLC25A11,* and* MDH2.*

### Metanephrine Measurements

Determination of urinary metanephrines was performed on 24-h urine samples. The samples were subjected to acid hydrolysis, neutralization, extraction, elution and finally analysed by high-performance liquid chromatography (HPLC) using the Chromsystems™ kit. Biochemical phenotypes were categorized as adrenergic, noradrenergic, or biochemically negative. The phenotype was defined adrenergic when the increment of metanephrine above the upper limits of normal exceeded 5% of the combined metanephrine and normetanephrine increments. Patients that did not fall within these criteria and with normetanephrine outputs above the upper limits of normal were classified as noradrenergic [23].

### Gas Chromatography–Mass Spectrometry (GC–MS) Analysis for Succinate Quantification

For succinate extraction, collected samples were thawed on ice. To 100 µL serum, 100 µL methanol (with ^13^C-succinate internal standard, Merck, catalogue number 491985), and 100 µL chloroform were added. Samples were then kept on ice for 10 min, vortexed, and centrifuged at 14,000 rpm for 10 min at 4 °C. The aqueous phase was recovered and subsequently dried (CentriVap, LabConco). The dried pellets were then stored at −80 °C. Subsequently, extracted metabolites were derivatized for 90 min at 37 °C with 10 µL of 40 mg/ml methoxyamine hydrochloride (Merck, #226,904) in pyridine (Merck). Then, 50 µl of N-tert-Butyldimethylsilyl-N-methyltrifluoroacetamide with 1% tert-Butyldimethylchlorosilane (Merck, #375,934) were added for 30 min at 60 °C. Extracted metabolites were analysed with an Intuvo 9000 GC/5977B MS System (Agilent Technologies Inc.) equipped with an HP-5MS capillary column (30 m × 0.25 mm × 0.25 µm). 1 µl of each sample was injected in splitless mode using an inlet liner temperature of 240 °C. GC runs were performed with helium as carrier gas at 1 ml/min. The GC oven temperature ramp was from 70 °C to 280 °C. The temperature of 70 °C was held for 2 min. Then, the first temperature ramp was from 70 °C to 140 °C at 3 °C/min. The second ramp was from 140 °C to 150 °C at 1 °C/min. The third temperature ramp was from 150 °C to 280 °C at 3 °C/min. The measurement of metabolites was performed under electron impact ionization at 70 eV. The ion source and transfer line temperature were set to 230 °C and 290 °C, respectively. Succinate was detected using a selected ion monitoring method. Three masses for succinate (215, 289, 331 mass to charge ratio or m/z) and one mass for succinate labelled + 4 (293 m/z) were detected at a retention time of 36.6 min. MS Quantitative Analysis software (Agilent Technologies Inc., version 10.2) was used for data analysis. For determination of succinate abundance, the integrated signal at m/z 289 was normalized by the integrated signal of the succinate labelled + 4. The absolute quantification of succinate was calculated by using a standard curve.

### Statistical Analysis

Statistical analysis was conducted using IBM SPSS Statistics™ version 29.0 (SPSS Inc.). Descriptive statistics are presented as frequencies and percentages for categorical variables, means and standard deviations for continuous variables with a parametric distribution, and medians with interquartile ranges for continuous variables with a non-parametric distribution. Normality was assessed using the Shapiro–Wilk W test. Differences between groups for independent non-parametric variables were evaluated using the Mann–Whitney U test, while differences for independent parametric variables were assessed using the t-test. Comparisons of categorical variables were performed using Pearson’s χ2 test or Fisher’s exact test when appropriate. Logistic regression analysis was conducted to evaluate potential relationships among significant variables. A p-value < 0.05 was considered statistically significant. Additionally, ROC curve analysis was performed to assess the accuracy of succinate levels in identifying lesions associated with Cluster 1 and metastatic disease. The relative confidence intervals of area under the ROC curve (AUROC) have been calculated by bootstrapping. Optimal cut-off values were determined based on the Youden index, and odds ratios with confidence intervals were calculated to quantify the predictive value for malignancy and genetic clustering. Binary logistic regression was also performed to assess the likelihood of Cluster 1 membership, using age at diagnosis, presence of metastases, and succinate levels as covariates. A multivariable analysis was conducted to examine the relationship between succinate levels and biochemical phenotype while adjusting for age at diagnosis, presence of metastases, and tumour size. Furthermore, to develop a predictive scoring system for metastatic disease, we divided continuous variables at optimal cut-off values associated with presence of metastasis determined through ROC analysis to obtain dichotomic variables. The scoring system was then constructed by normalizing odds ratios and iteratively refining variables to maximize predictive accuracy. To assess differences between our Preoperative Succinate MetAstatic Risk Tool (P-SMART) and ASES scoring system performances we confronted their AUROC through Delong test.

## Results

### Population Description

We enrolled 70 patients, comprising 41 females (58.6%) and 29 males (41.4%), with a mean age at diagnosis of 51.86 ± 16.27 years. Patients were divided based on their genetic cluster. In particular, 14, 10 and 46 patients were categorized as Cluster 1, Cluster 2 and negative for pathogenetic variants (NPV), respectively. No patient was categorized as Cluster 3. In our cohort, none of the patients showed multifocal primary disease. Moreover, only patients with bone or distant lymph node lesions confirmed bioptically have been considered metastatic.

**Fig. 1:**
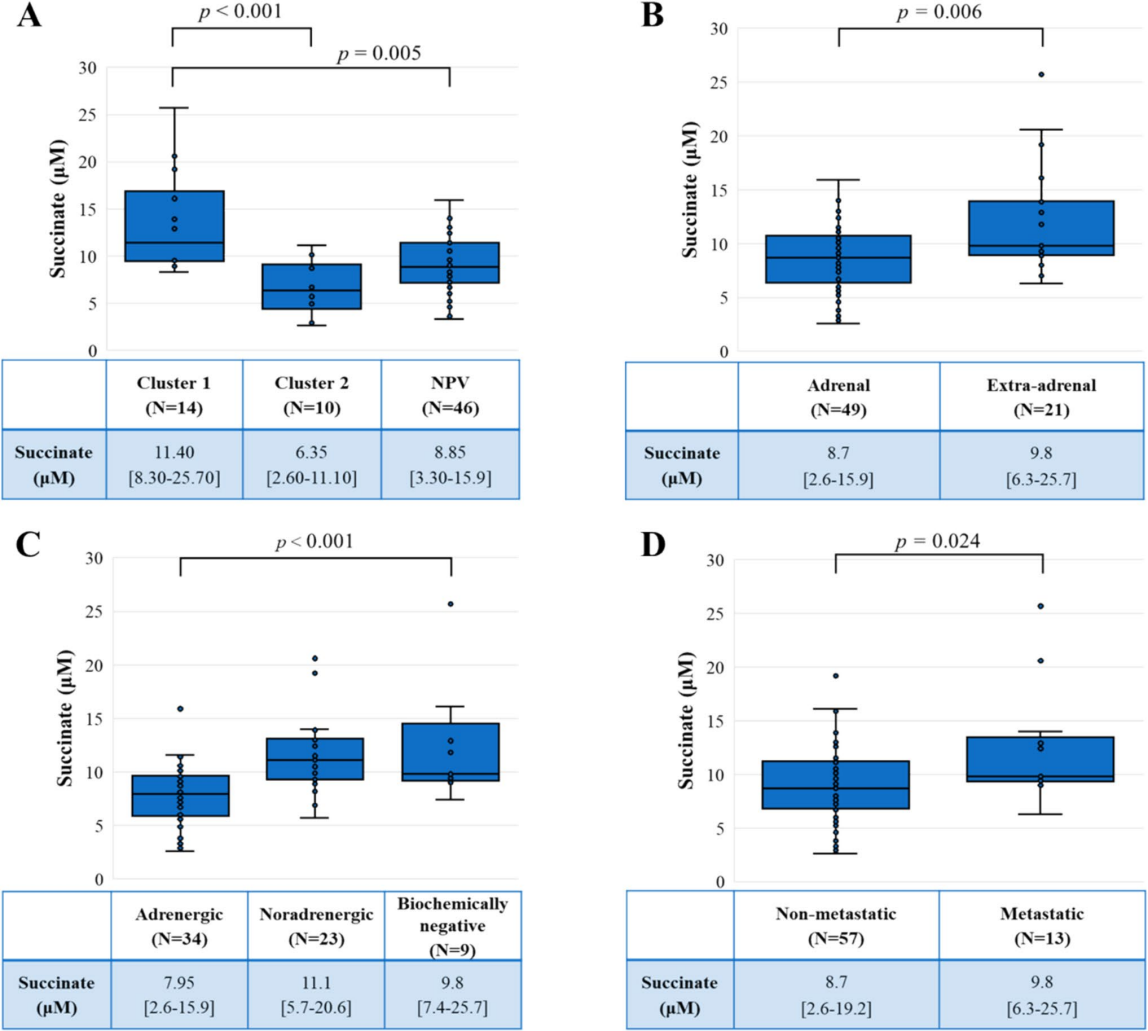
Comparison of succinate concentrations between different subgroups. Figure 1A: Comparison of succinate concentrations between Cluster 1, Cluster 2 and negative for pathogenic variants (NPV) patients. N = 14 Cluster 1, N = 10 Cluster 2, N = 46 NPV. Figure 1B Comparison of succinate concentrations between patients with adrenal and extra-adrenal PGLs. N = 49 adrenal, N = 21 extra-adrenal. Figure 1C: Comparison of succinate concentrations between biochemical phenotypes. N = 34 adrenergic, N = 23 noradrenergic, N = 9 biochemically negative. Figure 1D: Comparison of succinate concentrations between patients with non-metastatic and metastatic PGLs. N = 57 non-metastatic, N = 13 metastatic. Individual values are represented as blue circles. In the table, succinate concentrations (µM) are expressed as median [min–max]

Table 1 summarizes the relevant characteristics of the study population. Significant differences among groups were observed in terms of age at diagnosis (p < 0.001), TNM staging (p = 0.018), tumour dimension (p = 0.046), and urinary metanephrine outputs (p < 0.001). Cluster 1 patients were younger than those negative for pathogenic variants (NPV, p < 0.001). They had larger primary tumours compared to patients in Cluster 2 (Cluster 1 *vs* Cluster 2, p < 0.001), presented with more advanced AJCC TNM stage (Cluster 1 *vs* Cluster 2, p = 0.010 and Cluster 1 *vs* NPV, p = 0.018) and had lower outputs of urinary metanephrine than those in Cluster 2 or NPV (Cluster 1 *vs* Cluster 2, p < 0.001, and Cluster 1 *vs* NPV, p < 0.001). Other pathological data are available in Supplementary Table 1. Focusing on succinate concentrations, significant differences were observed among groups p < 0.001). In particular, Cluster 1 patients showed higher levels of succinate than the rest of the sample. (Fig. 1A).

**Table 1:** Population characteristics by cluster

		Cluster			Sign.		Sign.	Sign.	
		1	2	NPV	Overall		Cl.1 vs Cl.2	Cl.1 vs NPV	
Total	N	14	10	46	-		-	-	
Females	N (%)	9 (64.3)	6 (60.0)	26 (56.5)	0.871		-	-	
Age (years)	Mean (SD)	34.2 (12.6)	42.9 (13.4)	59.2 (12.4)	<0.001		0.334	<0.001	
Metastatic disease	N (%)	5 (35.7)	0 (0.0)	8 (17.4)	0.080		**-**	-	
AJCC TNM stage (Excluding n=4 HNPGLs)	N (%)				0.018		0.01	0.018	
I		1 (9.1)	8 (80.0)	24 (53.3)					
II		7 (54.5)	2 (20.0)	13 (28.9)					
III		1 (9.1)	0 (0.0)	0 (0.0)					
IV		3 (27.3)	0 (0.0)	8 (17.8)					
Dimensions (mm)	Mean (SD)	61.1 (27.8)	38.2 (25.8)	49.8 (28.7)	0.046		0.013	0.104	
Urinary metanephrine (μg/24h)	Median [min-max]	118 [45–150]	793 [98–4620]	925.5 [10–15945]	<0.001		0.002	<0.001	
Urinary normetanephrine (μg/24h)	Median [min-max]	1250 [115–14375]	484 [341–3400]	1385 [186–44574]	0.147		-	-

Regarding primary lesion location, 49 patients (70.0%) had an adrenal PGL, 16 (22.9%) had an extra-adrenal abdominal PGL, four had an HNPGL (5.7%), and one patient had a thoracic PGL (1.4%). Table 2 presents data stratified by primary tumour location, comparing adrenal PGLs (N = 49) with extra-adrenal PGLs (N = 21, including abdominal, thoracic, and head and neck PGLs). Extra-adrenal PGLs were more frequently associated with metastatic disease, larger lesions and lower outputs of urinary metanephrine (all p < 0.001). Furthermore, extra-adrenal lesions exhibited significantly higher succinate levels (extra-adrenal = 9.8 µM [6.3–25.7] *vs* adrenal = 8.7 µM [2.6–15.9], p = 0.006) (Fig. 1B). Excluding HNPGLs (N = 4) and considering only adrenal and thoraco-abdominal PGLs (N = 66), extra-adrenal PGLs showed more advanced AJCC TNM stage at diagnosis (p < 0.001). They also had larger lesions (extra-adrenal = 60 mm [30–150] *vs* adrenal = 40 mm [15–120], p = 0.005), lower urinary metanephrine outputs (extra-adrenal = 126 µg/day [10–1652] *vs* adrenal = 1107.5 µg/day [44–15945], p < 0.001), and higher succinate levels (extra-adrenal = 9.8 µM [6.3–25.7] *vs* adrenal = 8.7 µM [2.6–15.9], p = 0.012).

**Table 2:** Population characteristics by primary localization

		**Primary localization**		**Sign**
		**Adrenal**	**Extra-adrenal**	
Total	N	49	21	-
Females	N (%)	29 (59.2)	12 (57.1)	1
Age (years)	Mean (SD)	53.59 (15.23)	47.81 (34.33)	0.172
Metastatic disease	N (%)	3 (6.1)	10 (47.6)	** < **0.001
AJCC TNM stage (Excluding n = 4 HNPGLs)	N (%)			< 0.001
I		32 (65.3)	1 (5.9)	
II		14 (28.6)	7 (41.2)	
III		0 (0.0)	1 (5.9)	
IV		3 (6.1)	8 (47.1)	
Dimensions (mm)	Mean (SD)	41.8 (21.97)	71.1 (33.1)	0.001
Urinary metanephrine (μg/24 h)	Median [min–max]	2196 [44–15945]	124.5 [10–1652]	< 0.001
Urinary normetanephrine (μg/24 h)	Median [min–max]	1108 [298–44574]	866 [115–14375]	0.163

Considering the biochemical phenotype (adrenergic, noradrenergic, and biochemically negative), a significant difference was found among groups regarding the presence of metastases (3/34 [8.8%], 4/23 [19.9%], and 6/9 [66.7%] respectively, p < 0.001) and succinate levels (7.95 [2.6–15.9], 11.1 [5.7–20.6], and 9.8 [7.4–25.7] respectively, p < 0.001) (Fig. 1C). In multivariable regression, after adjusting for age at diagnosis, presence of metastases, and tumour size and using succinate level as the dependent variable, patients with a noradrenergic phenotype had significantly higher succinate levels than those with adrenergic phenotype (p = 0.002) without differences with biochemically negative patients (p = 0.647). Additionally, after adjusting for mutational status, lesion dimension and localization, succinate levels are independently associated with the presence of metastasis (p = 0.039).

When further dividing the population considering the presence of metastases, metastatic patients had larger primary tumours (p = 0.003) and lower metanephrine outputs (p = 0.037) (Table 3). In addition, metastatic patients exhibited higher succinate levels than the non-metastatic ones (9.8 µM [6.3–25.7] *vs* 8.8 µM [2.6–19.2], respectively, p = 0.024) (Fig. 1D).

**Table 3:** Population characteristics by disease status

		**Non-metastatic**	**Metastatic**	**Sign**
Total	N	57	13	-
Females	N (%)	35 (61.4)	6 (46.2)	0.361
Age (years)	Mean (SD)	52.18 (15.54)	50.46 (19.80)	0.815
Extra-adrenal localization	N (%)	11 (19.3)	10 (76.9)	0.001
Dimensions (mm)	Mean (SD)	44.09 (22.27)	78.55 (38.45)	**0.003**
Urinary metanephrine (μg/24 h)	Median [min–max]	744 [44–15945]	140 [10–5193]	**0.037**
Urinary normetanephrine (μg/24 h)	Median [min–max]	1110 [115–44574]	768 [186–5572]	0.249

**Fig. 2:**
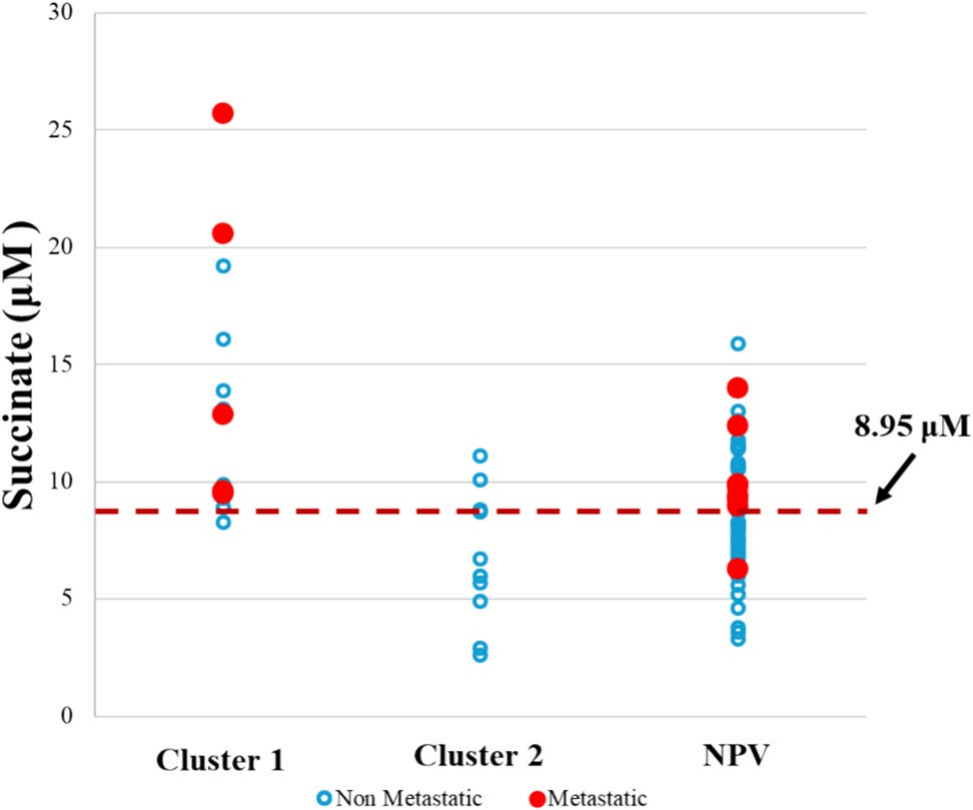
Distribution of succinate concentrations per cluster. A cut-off of 8.95 μM (dotted line) correctly classifies 100% of metastatic patients belonging to Cluster 1 and 85.7% of metastatic patients with negative genetic test results

ROC curve analysis showed that a succinate level ≥ 8.95 µM predicted metastatic disease with a sensitivity of 92.3% and a specificity of 56.1% (AUC 0.701 ± 0.073, 95% CI: 0.558–0.844, p = 0.006, Youden index 0.484). Additionally, a succinate level ≥ 8.95 µM significantly increased the likelihood of metastases (OR = 4.928, 95% CI: 1.222–19.873, p = 0.029). Notably, this threshold correctly classified all metastatic patients in Cluster 1 and 85.7% of metastatic patients with negative genetic test results (Fig. 2). After excluding HNPGLs, ROC analysis showed similar results. In fact, a succinate level ≥ 8.95 µM remained the optimal threshold for predicting a metastatic disease, with a sensitivity of 90.9% and specificity of 56.4% (AUC 0.698 ± 0.080, 95% CI: 0.542–0.854, p = 0.013, Youden index 0.473), significantly increasing metastasis risk (OR = 12.917, 95% CI: 1.545–107.996, p = 0.006). Furthermore, a succinate level ≥ 8.95 µM was also able to predict pathogenic variants included in Cluster 1 with a sensitivity of 85.7% and a specificity of 55.4% (AUC 0.786 ± 0.063, 95% CI: 0.663–0.908, p < 0.001, Youden index 0.411). Binary regression analysis using Cluster 1 versus the rest of the sample as dependent variable and age at diagnosis, presence of metastases, and succinate level ≥ 8.95 µM as covariates showed that a succinate level ≥ 8.95 µM significantly increased the probability of any pathogenic variant included in Cluster 1 (OR = 10.20, 95% CI: 1.28—83.33, p = 0.028). Considering metastatic patients, ROC analysis identified that a succinate level ≥ 9.45 µM is predictive of belonging to Cluster 1 lesions, with a sensitivity of 100% and specificity of 50.0% (AUC 0.775 ± 0.138, 95% CI: 0.505–1.045, p = 0.046, Youden index 0.500). Across the entire cohort, a succinate level ≥ 9.45 µM significantly increased the likelihood of Cluster 1 classification (OR = 5.667, 95% CI: 1.419–22.630, p = 0.015).

### Development of the P-SMART Scoring System

To refine malignancy prediction and to develop a discrete scoring system, we further analysed variables, categorical or continuous, associated with metastases. Due to the limited sample size, HNPGL cases were excluded from this analysis. A score using only continuous variables is described in supplementary material (Supplementary Fig. 1).

We constructed a multivariable logistic regression model (not shown) incorporating succinate, lesion size, and urinary metanephrine as continuous variables, along with tumour location as a categorical variable.

To develop a discrete, point-based score, continuous variables demonstrating independent association with metastasis (lesion size, serum succinate) were dichotomized as dummy variables (0/1) using optimal cut-off values determined through ROC curve analysis (not shown). The resulting dummy variables along with the categorical parameters [i.e. extra-adrenal primary localization (no/yes) and adrenergic vs non-adrenergic phenotype (0/1)] were introduced in iterative binary regression models to confirm their independent association with metastasis and to obtain relative odds ratios for metastasis (Table 4). Points were assigned proportionally to the odds ratios associated with each parameter, normalized to the lowest OR (Table 4). To optimize the score, we iteratively removed each variable from the score calculation to identify the variables not essential to model accuracy, in particular, removing adrenergic vs non adrenergic phenotype did not affect the consistency of model accuracy.

**Table 4:** Unadjusted and normalized odds ratios for metastasis per metastasis-associated variable

Parameter	OR for metastasis	
	**Unadjusted**	**Normalized**
Extra-adrenal localization	13.63	3
Succinate ≥ 8.95 µM	15.36	3.5
Lesion size ≥ 7.0 cm	14.00	3
Non-adrenergic phenotype	4.70	1

### P-SMART Scoring System

Our final scoring system, which we named P-SMART (Presurgical Succinate MetAstatic Risk Tool), assigns: 3 points for extra-adrenal localization, 3.5 points for serum succinate > 8.95 µM, and 3 points for lesion size ≥ 7.0 cm. We didn’t find statistically significant difference (DeLong test *p* = 0.850) between the first model yielded an AUC of 0.85 (95% CI: 0.73–0.97), comparable to the performance of the P-SMART score (AUC = 0.87).

**Fig. 3:**
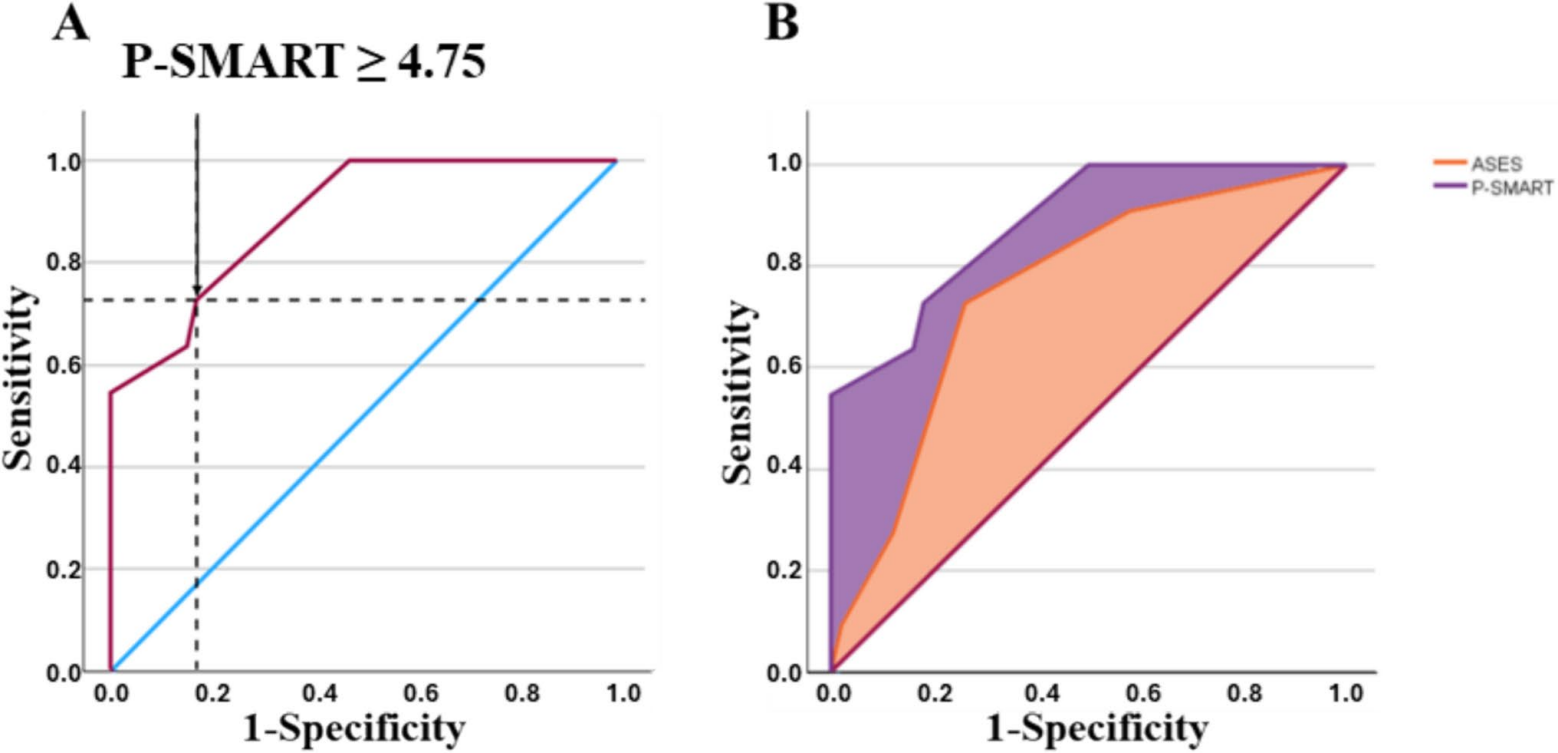
ROC curve analysis for metastasis. Figure 3A: ROC curve analysis for metastasis considering a P-SMART score ≥ 4.75 (AUC 0.885 ± 0.052, 95% CI: 0.782–0.987,* p* < 0.001, Youden index 0.547). A P-SMART score equal or higher than 4.75 can identify the presence of metastasis with a sensitivity of 72.7% and specificity of 82%. Figure 3B: Graphic comparison between P-SMART (purple) and ASES (orange) ROCs (AUC 0.885 [0.782–0.987] vs 0.752 [0.600–0.903]; DeLong test’s p = 0.005)

ROC analysis demonstrated that a score higher than 4.75 predicted metastatic disease with a sensitivity of 72.7% and specificity of 83% (AUC 0.891 ± 0.050, 95% CI: 0.794–0.988, p < 0.001, Youden index 0.557) (Fig. 3A).

In our dataset ASES score equal or higher than 2 (according to the published cut-off [17]) predicted metastatic disease with 72.7% sensitivity and 74% specificity (AUC 0.752 ± 0.077, 95% CI: 0.600–0.903, p = 0.001). Comparing the AUROC through DeLong test, we found a significant superiority in predicting metastases for P-SMART as compared to ASES scoring (AUC 0.891 [0.794–0.988] *vs* 0.752 [0.600–0.903]; p = 0.005) (Fig. 3B).

## Discussion

In this study, we investigated the potential of succinate as a diagnostic and pre-surgical prognostic marker in patients affected by PGLs. By combining succinate levels with tumour size and primary location, we developed the P-SMART (Preoperative Succinate, MetAstatic Risk Tool), a new scoring tool that shows good accuracy in predicting the presence of metastatic lesions and was even superior to ASES scoring.

We confirm [1] that almost 40% of PGL patients carry a germline pathogenic variant in one of the susceptibility genes, therefore necessitating lifelong patient monitoring. Hence, minimally invasive, reliable and safe monitoring tools for longitudinal follow-up in PGL patients are required. Among the biomarkers, succinate has demonstrated good performances for diagnosing and monitoring individuals with pathogenic variants in *SDH* genes. Recent studies have shown that succinate can effectively differentiate asymptomatic *SDHB* variant carriers from non-carriers and identify individuals who have developed the disease, with succinate levels correlating with metastatic disease extent. Accordingly, Bancel et al. [24] proposed the succinate-to-fumarate ratio as a method to improve the accuracy of detecting *SDHx* pathogenic variants and associated lesions, allowing for individualized imaging surveillance.

Measuring succinate is a low-cost, minimally invasive, serum-based method and may be particularly valuable for patients with negative conventional biomarkers such as metanephrines, for whom imaging remains the only available monitoring option, increasing healthcare costs and imaging-associated risks.

In this study we also addressed the current lack of single, highly accurate biomarker able to identify patients at higher risk of developing metastases. There are several established risk factors that help recognize patients requiring closer follow-up. They include younger age, extra-adrenal location, larger lesions (> 5 cm), *SDHB* pathogenic variants and less differentiated biochemical phenotypes. In addition, metastatic risk scores (such as PASS and GAPP) are already established [14], but they rely on post-surgical histological parameters. Identifying novel, pre-surgical parameters could help identify which patients could benefit from more intensive diagnostic methods, like functional imaging. Current biochemical markers, including plasma or urinary metanephrines and chromogranin A, a blood non-specific neuroendocrine tumour marker, have limitations, especially in biochemically negative tumours. Additionally, metastatic disease may be less differentiated than the primary tumour, potentially leading to non-functional tumours. This study aimed to evaluate the applicability of succinate measurement as a potential biomarker in PGLs within our cohort, including patients with pathogenic variants beyond *SDHB* and those with metastatic disease. In line with the existing literature, our study confirms that patients with pathogenic variants in Cluster 1, noradrenergic or biochemically negative phenotypes, and extra-adrenal lesions are at higher metastatic risk [25]. Furthermore, our findings corroborate previous studies [24], showing higher succinate levels in patients with Cluster 1 pathogenic variants as compared to those within Cluster 2 or those with negative genetic analyses. The observed higher succinate levels in extra-adrenal lesions may be linked to the higher prevalence of these locations in Cluster 1 patients, as reported in other studies [6]. We also explored succinate as a potential screening tool for metastatic lesions, identifying a cut-off of 8.95 μM to distinguish metastatic from non-metastatic patients. Importantly, although succinate accumulation is often linked to SDH dysfunction, the multivariable analysis showed that elevated succinate levels independently predicted metastatic disease, even when adjusted for tumour size and extra-adrenal localization. This suggests that succinate provides additive predictive value beyond these established factors. Moreover, succinate levels remained significantly associated with metastatic status even after adjusting for mutational status, suggesting that succinate elevation could be linked to tumour aggressiveness beyond genetic background alone.

Recognizing that succinate alone may not be sufficiently predictive, we developed a novel composite score incorporating pre-operatively accessible parameters: succinate levels (μM), lesion size (cm), and log [urinary metanephrine mcg/24 h], finding a threshold of 7.33 with improved diagnostic performance.

We further analysed our data finally developing the P-SMART, combining succinate levels with tumour dimension and localization, which exhibited good accuracy in predicting the presence of metastatic lesions. A comparable pre-operative scoring system (ASES) [17] yielded lower predictive performance in our dataset as revealed by DeLong test. It is worth noting that a recent important contribution by Pamporaki et al. [26] developed and externally validated machine learning-based models for predicting metastatic PPGL using a large international cohort of 788 patients, achieving superior performance (AUC: 0.942, sensitivity: 83%, specificity: 92%). This model and the related one developed by Ronchi’s group [27] represent powerful and generalizable approaches. It will be interesting to compare the diagnostic performances of these models with that of P-SMART.

Our findings support two primary conclusions. First, as suggested by other authors [22], succinate represents a potential non-invasive and cost-effective biomarker valuable for both initial diagnosis and routine follow-up of *SDHx*-associated lesions, particularly for biochemically negative or dopamine-producing tumours where plasma 3-methoxythyramine measurement is unavailable. Second, pre-operative succinate levels can contribute to the initial assessment of PGLs, potentially indicating Cluster 1 pathogenic variant association and metastatic risk, thereby optimizing the use of second-line diagnostic imaging such as nuclear medicine. As highlighted in a recent review by Timmers et al. [28] whole-body imaging should be prompted by the discovery of an extra-adrenal PGL, an adrenal PGL larger than 5 cm or by a noradrenergic or dopaminergic phenotype. Succinate measurement could further refine these indications, avoiding excessive radiation exposure, financial burden, and treatment delays. Additionally, longitudinal succinate monitoring could enhance the precision of imaging strategies during follow-up, potentially reducing healthcare costs and patient exposure to unnecessary diagnostic procedures. This study presents some limitations including (i) a limited sample size, especially among Cluster 1 and metastatic patients, precluding subgroup analysis; (ii) absence of a validation cohort, necessary to assess the robustness of the score; (iii) population heterogeneity; (iv) limited length of follow up, and (v) the retrospective cross-sectional design preventing conclusions on metastatic risk prediction or longitudinal disease evolution. Additionally, complete pathological data were not uniformly available across our cohort reducing our ability to evaluate associations between pathological features and genotype or metastatic risk. Despite this, these results are promising. Succinate measurement, particularly when integrated into a multi-parametric score incorporating tumour dimension and localization, may aid in identifying patients with metastatic or high-risk PGLs. Further prospective studies with larger cohorts are warranted to validate our findings and refine the clinical applications of the P-SMART score.

## Supplementary Information

Below is the link to the electronic supplementary material.

ESM 1 (DOCX 72.2 KB)

## Data Availability

No datasets were generated or analysed during the current study.
