# Correction: Presurgical Succinate MetAstatic Risk Tool (P-SMART) in Paragangliomas

**DOI:** 10.1007/s12022-025-09884-x

**Published:** 2025-10-22

**Authors:** Elena Rapizzi, Lorenzo Zanatta, Alice Santi, Fabio Staderini, Niccolò Galeotti, Tonino Ercolino, Francesca Amore, Clotilde Sparano, Mario Maggi, Letizia Canu

**Affiliations:** 1 Dept. Experimental and Clinical Medicine, University of Florence, Florence, Italy; 2 Centro Di Ricerca E Innovazione Sulle Patologie Surrenaliche, AOU Careggi, Florence, Italy; 3 ENS@T Centre of Excellence, Florence, Italy; 4 Dept. Experimental and Clinical Biomedical Sciences, University of Florence, Florence, Italy; 5 Careggi University Hospital, Florence, Italy

**Correction: Endocrine Pathology (2025) 36:33**

10.1007/s12022-025-09878-9

The original publication of this article was incorrectly published with structured abstract. The abstract should be unstructured. The original article has been corrected.

