# Supplementary material for: Presurgical Succinate MetAstatic Risk Tool (P-SMART) in Paragangliomas
Source: Endocr Pathol. 2025 Sep 30;36(1):33. doi: 10.1007/s12022-025-09878-9 (PMC12484271; doi:10.1007/s12022-025-09878-9)
Supplement: Supplementary file 1 — (DOCX 72.2 KB) [file 12022_2025_9878_MOESM1_ESM.docx]

|  |  | **Cluster** | | | | **Sign.** |
| --- | --- | --- | --- | --- | --- | --- |
|  |  | **1** | **2** | **NPV** | **Overall** | |
| **Total** | N | 7 | 9 | 37 | - | |
| **Vascular invasion** | N (%) | 1 (14.3) | 2 (22.2) | 5 (13.5) | - | |
| **Ki67 index >3%** | N (%) | 2 (28.6) | 3 (33.3) | 4 (10.5) | - | |
| **Total** | N | 7 | 7 | 25 | - | |
| **Necrosis** | N (%) | 0 (0.0) | 1 (14.3) | 2 (8.0) | - | |

**Supplementary Table 1:** Other histopathological data available for patients, divided by cluster. Necrosis was available for 39 patients. S100 staining was available for 5 patients only (positives: 1/1 in Cluster 1 patients, 1/1 in Cluster 2 patients, 2/3 in NPV patients). Even confronting subgroups (Cl1 vs Cl2 or Cl1 vs NPV) no differences were found.


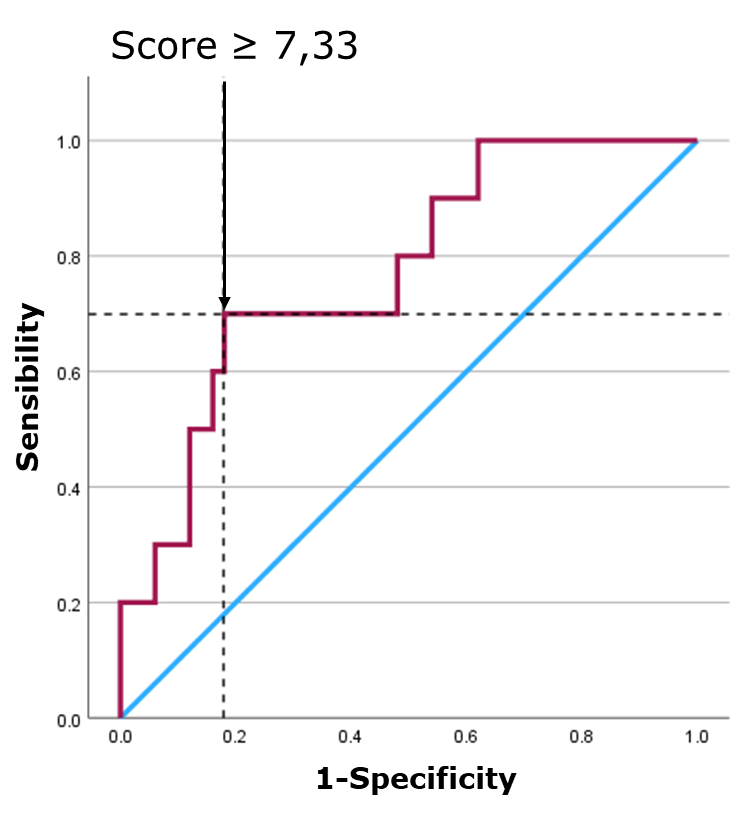


**Supplementary Figure 1:** combining the continuous variables significantly associated with metastases, such as succinate concentration, primary lesion size (cm), and MN levels (mcg/24h, log transformed), we developed another scoring system. $Score=\frac{Succinate \left( \mu M \right)+primary lesion size (cm)}{\log[MN\left( \frac{\mu g}{24h} \right)]}$

ROC curve analysis determined that a score ≥ 7.33 predicted metastatic disease with a sensitivity of 70% and specificity of 82% (AUC 0.772 ± 0.078, 95% CI: 0.619-0.925, p < 0.001, Youden index 0.520).
